# Supplementary material for: Developmental changes in straight gait in childhood
Source: PLoS One. 2023 Feb 9;18(2):e0281037. doi: 10.1371/journal.pone.0281037 (PMC9910736; doi:10.1371/journal.pone.0281037)
Supplement: Attachment — Submitted filename: Response to Reviewers.docx [file pone.0281037.s002.docx]

**We thank the reviewers for the meticulous review of our manuscript and the thoughtful comments. We have tried our best to address all the points raised. We have provided point-by-point responses to the reviewers’ comments. In the manuscript, the modified parts are shown in red.**

**Response to Reviewers:**

**Reviewer #1:**

The authors state they developed both indices but it is not clear if the P-index is as previously used in studies?
I have concerns regarding the G index as a measure of stability. The p-index makes sense and is a measure of body sway from the direction of progress.
The G-index than also incorporates deviation between the direction of progress and the target direction. The authors conclude that pace and stability may contribute to the g-index. However, as mentioned by the authors this measure also "reflects the ability to accurately focus on the target...". This potentially incorporates a large array of variables that have not been considered, some of which may also change with age (attention, ability to follow commands, eye-sight, other distractions in the room etc..). Its not clear from the Methods what instructions were given, how definite these instructions were and what the target was. All of this may impact of the g-index. it is also not clear what this index adds in comparison to just looking at the deviation of one, appropriate VICON marker from the A-P axis for example?

**Response:** We deeply appreciate the valuable comments of the reviewer. As far as we know, there is no previous research focused on the P index, but we believe that this should be a completely novel index. The G index is as described in the manuscript and is based on previous research. As you suggest, the G-index also incorporates the deviation between the direction of travel and the direction of the target, and is influenced by various factors. We have modified the purpose and hypothesis of this research more clearly (Line 102), and added detailed instructions to the methods section (Line 127).

It is clear that various factors are involved in postural control. Therefore, it is required to evaluate straight progress including that element. We believe that it is not possible to consider various factors of attitude control by simply looking at the deviation of one suitable VICON marker from the AP axis.

other points:

Abstract:

Line 30: typically developing might be better than normally developing

**Response**: Thank you for your advice. As you pointed out, I changed it to “typically”. (Line 30)

Line 39: CV not defined on first use

**Response:** Thank you for pointing this out. As you said, I defined the first "CV". (Line 39)

Introduction

Line 59: Need a reference for statement in first sentence

**Response:** Thank you very much for your pointing out. I decided that this part was not appropriate and deleted it.

Line 69: Spell error- 'clearly'

**Response:** Thank you very much for your pointing out. This has been corrected accordingly. (Line 63)

Line 77 and line 69: I think 'meandering gait' and 'straight gait' need to be more clearly defined and then use one term consistently (if they are same thing?)

**Response:** Thank you for your appropriate remarks. Since "meandering" and "straight" are confusing, I decided to use the term "straight gait" as a unified term.

Methods:

more information needed on instructions and gait procedures given the young population and that walking towards target is one of the measured variables. How much instruction? What target? etc..

**Response:** The explanation was certainly inadequate. As you pointed out, I added detailed instructions to methods (line 127).

The middle 4 meters was recorded but then 2m was used for defining straight. Why only 2/4 and which 2?

**Response:** Due to the experimental environment, we had no choice but to limit the analysis range for defining straight movement to 2m. The reason is that the maximum range for recording all marker data of all participants was 2m. I wrote about it in Line 148.

Line 159: HC is presumably heel-contact but needs to be defined on first use

**Response:** Thank you for your advice. ``Heel contact'' was added. (Line 150).

Line 195: don't think SD is defined on first use

**Response:** This has been defined in introduction section (Line 93).

Table 3: DEfine SV in legend (or table). p<0.05 is shown for differences compared to adult group but not clear if there were differences between other groups also or not?

**Response:** Thanking for pointing that out. It is indicated as “Step velocity” in the table. In addition, I added below the table that there was no difference from the non-adult group.

Table 4: difficult to follow with all abbreviations and full variable names could easily be included

**Response:** Thanks to your point, I defined the abbreviations other than SI and CV in the table, and defined SI and CV below the table.

Discussion

Line 282: Am not sure it can be said that SV, SW etc contribute to the XCOM variables? They correlate with them which is different. This is maybe a bigger point that needs discussion and is a concern. The two XCOM variables are correlated with a host of other variables all (or at least many) of which are possibly age related so a correlation is not surprising and not sure what it tells us? If XCOM-G and XCOM P were correlated with height for example there may well be a correlation but that just reflects that both change with age/growth?

**Response:** Thank you for your very important remarks. Our previous study (Mani et al., reference [7]) reported that individual gait variables such as SV and SW were not perfectly correlated with age, indicating different developmental characteristics. We thought that by examining the relationships between these five gait functional domains and the straight gait indices could help us understand the kind of control functions that are required for straight gait.

In this study, we showed that the ability for straight gait develops with age (Table 3) and further showed the gait variables that are associated with two indices of straight gait (Table 4).

**Reviewer #2:**

About positioning and rationale of the study

**Response:** Thank you for your very useful remarks. Certainly, the link between DCD and meandering gait is confusing. So, we decided to remove the content of the first paragraph of the original manuscript from the revised manuscript because it was less relevant to this study. Instead, we clarified the motivation for this study by citing previous studies on the development of gait stability.

**The purposes and hypotheses are confusing, particularly purpose 2.**

**Response:** Thanks for pointing it out. It's certainly confusing, so I changed the hypothesis from 3 to 2 (Line 103). Then, I revised the two hypotheses so that they were clearly stated in the discussion (Line 262 and 282).

Methods

The use of the XCOM measures for a whole trial (multiple gait cycles) as opposed to within step appears to be different to the intended use by Hof (2005) and Hallemans et al. (2018), this is not clear within the introduction and has not been fully justified within the manuscript.

**Response:** Thank you for pointing this out. This study was carried out using a modified method, referring to references 6 and 7. Since there may be an effect of arranging it a little, I added it as a research limit (line 330).

The descriptions of the variables XCOMg and XCOMp describe an integral, however this is not apparent in Figure 1. It looks like you have tried to take a single point in time as an example, but the figure could be improved and/or described properly. Furthermore the ‘Goal axis’ should be added to this figure along x = 0.

**Response:** Thank you for your advice. It is true that Fig1 is difficult to understand, so I corrected Fig1 and added the explanation of the figure, referring to what you pointed out.

The latter descriptions of these indies on line 277-279 are far clearer than any earlier description so I would suggest using this terminology for the method and the figure to support the understanding of the terms.

**Response:** Thank you for your advice. Based on your advice, I decided to add the corresponding part to the introduction. (Line 80)

The variables were defined over a period of 2 m with at least 2 gait cycles recorded:

How were these selected from the 6 m walkway?

Due to the evident range in COM position and XCOM over the gait cycle (from figure 1), should the number of gait cycles analysed for each individual have been standardised?

**Response:** Thank you for your advice. The maximum range for recording all marker data of all participants was 2m. We were concerned that the measurement range (distance) of each subject would change if we used the walking cycle; hence, we decided to analyze by setting the distance to 2 m. This has been described in Line 148. Normalization was done for each individual's COM height.

Results, discussion and conclusions

The Results section is clear with well-presented tables, but would benefit from a statement of results prior to the table introduction.

Again the discussion section would benefit from an introductory sentence or two which summarise the study findings prior to a sub-heading.

**Response:** Thank you for your useful advice. I added a short heading on line 220 as per your advice.

Line 257: is this supported by both of these variables or XCOMg only?

I don’t think the second and third hypothesis are addressed directly in the discussion.

The statement which concludes your paper: “The two indices presented in this study are effective parameters for evaluating meandering and postural stability during gait”. This feels like it requires further justification and presentation of data. To link with the introduction, gait which is defined as meandering should have been assessed and defined using your two parameters to compare to gait which isn’t meandering.

**Response:** Thank you for your comment. The first sentence of the discussion indicated two variables. However, I thought the original text was difficult to understand, so as you pointed out, I decided to consider each hypothesis separately. As shown earlier, the hypotheses were changed into two (Line 103), and the discussion was modified to link with the introduction (Line 262 and 283).

Minor comments:

Line 115: suggest ‘reduces to’

**Response:** Thank you for your advice. I changed it as you pointed out (line 104).

Line 117: ‘involved’ doesn’t make sense her as a term

**Response:** Thank you for pointing this out. As you pointed out, I corrected the sentence to be appropriate for deletion. (Line 105).

119: what are control mechanisms?

**Response:** Thanks for your questions. This has been changed to “Control function.” This indicates an element related to postural control during walking, as shown by Mani et al.

Line 132: who provide written consent? The guardian or the child?

**Response:** Thank you for pointing this out. The guardians provided consent. This has been added accordingly (Line 120).

Line 158: two gait cycles per trial or in total?

**Response:** Thanks for your questions. It is two gait cycles per trial were recorded.

Line 220: I don’t think a % is an appropriate level and this should be reported as a decimal.

**Response:** Thank you for pointing this out. We have corrected this accordingly (Line 216).

Line 258: please clearly define which age groups this statement is true for.

**Response:** Thank you for pointing this out. We have clearly defined the relevant age group. (Line 263).

Table 1: sex should read ‘male’ or ‘female’

**Response:** Thank you for pointing this out. We have corrected this accordingly (Table 1).

Table 3: check title

**Response:** Thank you for pointing this out. I made some changes to the title.

Figure 1 title should define the two factors fully in the title.

**Response:** Thank you for pointing this out. I have modified Figure 1 and its description.
