# Supplementary material for: Developmental changes in straight gait in childhood
Source: PLoS One. 2023 Feb 9;18(2):e0281037. doi: 10.1371/journal.pone.0281037 (PMC9910736; doi:10.1371/journal.pone.0281037)
Supplement: Attachment — Submitted filename: Response to Reviewers.docx [file pone.0281037.s003.docx]

**Response to Reviewers**

We thank you for your peer review and comments on our second submitted manuscript.

We have done our best to address all points raised. Provided a point-by-point response to the reviewer's comments.

In the manuscript, the changed parts are shown in red.

**Response to Reviewers:**

**Reviewer #1**:

This sentence in Introduction is not clear-

changes in straight gait and the control mechanisms involved. We hypothesized that: (a) straight gait reduces to at younger ages and reaches the adult level around the age of 7 years [4];

is it "ability to walk in straight line is reduced at younger ages"?

→Thank you for your advice. We decided the first half of this sentence was confusing, so we fixed it. (Line 114)

Methods

the same position. After practicing the task several times, the results were recorded five times

Was a mean of the 5 trials then used for analysis or one representative?

→Thank you, you are right. We have added an explanation to Line 152.

**Reviewer #2:**

Introduction: Line 58-59: This still feels a little undefined and not fully justified as a reference to a theme/topic. Also the justification that there is not enough research feels very subjective. To link and lead onto the following paragraph there at least needs to be a description of the relevance of DCD to meandering gait.

→Thank you for your advice. We discussed the topic of walking in children with developmental disorders, and deepened the content so that the purpose of this research would be linked. (Line 58-69)

Line 86-88: this sentence needs rewording: I am not sure what it is trying to say, it doesn’t make sense.

→Thank you for pointing this out. We fixed the wording. (Line 96-98)

Line 104: hypothesis 1 needs rewording as ‘reduces to at younger’ does not make sense

→Thank you for your advice. We decided the first half of this sentence was confusing, so we fixed it. (Line 114)

Line 128: ‘calling’ would be better described as encouraging them forwards

→Thank you for pointing out. I have corrected it as you pointed out. (Line 138)

Line 129: was the walking path painted/drawn on the floor?

→Yes, you are correct. I added a photo (Fig 1). (Line 157-159)

Line 160/161: I think earlier this was defined as the position of the researcher? It may be better defined as the start and end point of the walking bouts? This might be better used throughout as opposed to the current reference as ‘start position to parents’ such as line 173 also.

→Thank you for pointing out. Sorry, this is the part I forgot to correct in the first review. I decided to define the start and end points of the walk as you suggested. (Line 150-151, 177 and 190)

Line 219: these statements feel highly cemented in the outcomes of the statistical tests and without any real consideration of magnitude of effect or what is a meaningful difference. IN Table 3 we can see substantial differences in XCOMG between Adults and 7-8 and 9-10 and I think this should be considered.

→Thank you for pointing out. Certainly, the values in the 7-8 years group and the 9-10 years group were still small compared to the adult group, which may be due to the growth process. However, it is true that there were no statistically significant differences between the 7-8years group compared to the adult group. So I mentioned it in the discussion, not the result.

Discussion: The XCOMG variable has been discussed with relevance to other data and studies but this has not been compared or contrasted in terms of numerical outcomes in the discussion.

→Thank you for your advice.Based on the results of comparison by age, we added what kind of abilities can be acquired by what age (Line 306-309).

In particular Hallemans

Please check the references: I am not sure that they align; Hallemans for example is 6 in the list and 7 in the text.

→Thank you for pointing this out. We rechecked and revised the reference list and manuscript.
